# Understanding Islet Autoantibodies in Prediction of Type 1 Diabetes

**DOI:** 10.1210/jendso/bvad160

**Published:** 2024-01-02

**Authors:** Xiaofan Jia, Liping Yu

**Affiliations:** Barbara Davis Center for Diabetes, University of Colorado School of Medicine, Aurora, CO 80045, USA; Barbara Davis Center for Diabetes, University of Colorado School of Medicine, Aurora, CO 80045, USA

**Keywords:** islet autoantibody, type 1 diabetes, prediction, antibody assay

## Abstract

As screening studies and preventive interventions for type 1 diabetes (T1D) advance rapidly, the utility of islet autoantibodies (IAbs) in T1D prediction comes with challenges for early and accurate disease progression prediction. Refining features of IAbs can provide more accurate risk assessment. The advances in islet autoantibodies assay techniques help to screen out islet autoantibodies with high efficiency and high disease specificity. Exploring new islet autoantibodies to neoepitopes/neoantigens remains a hot research field for improving prediction and disease pathogenesis. We will review the recent research progresses of islet autoantibodies to better understand the utility of islet autoantibodies in prediction of T1D.

Type 1 diabetes (T1D) is an autoimmune disease characterized by hyperglycemia and metabolic disorder, which is caused by the destruction of insulin-producing pancreatic β-cells and absolute insulin deficiency [1]. Individuals with T1D depend on external insulin treatment to maintain their blood glucose levels within an appropriate range, preventing or reducing the risk of acute complications such as diabetic ketoacidosis (DKA), severe hypoglycemia, and chronic vascular complications associated with diabetes. As of 2017, estimated global numbers of incident and prevalent cases of T1D were to be 234 710 and 9 004 610, respectively [2]. T1D can manifest at any age, but it typically develops during childhood [3]. The 10th edition of the International Diabetes Federation (IDF) Atlas estimates that 1 211 900 children and adolescents younger than 20 years old living with T1D globally [4]. Moreover, the incidence of T1D is on the rise worldwide, with an estimated 100 000 new cases diagnosed in children under 15 years of age each year [4].

As compelling evidence of the autoimmune pathogenesis of T1D, the presence of islet autoantibodies (IAbs) plays a crucial role in supporting the diagnosis of T1D [5-11]. Remarkably, 80% to 90% of childhood patients have detectable IAbs at the onset of T1D [12]. However, even with rapid and timely diagnosis and treatment, severe hyperglycemia and DKA are still associated with elevated mortality rates, particularly among younger patients [13]. By the time of diagnosis, 80% to 90% of β-cell mass has been lost [14], making it exceedingly challenging to intervene or reverse the progression of T1D at this advanced stage. The significant discoveries regarding the presence of IAbs preceding the clinical onset of T1D offer the tantalizing possibility of predicting and preventing the disease [15]. Accumulated evidence from longitudinal cohort studies and large-scale screening efforts for T1D has led to the establishment of 4 well-characterized IAbs, including autoantibodies targeting insulin (IAA), glutamic acid decarboxylase (GADA), insulinoma-associated antigen-2 (IA-2A), and zinc transporter 8 (ZnT8A), as key biomarkers for prediction of type 1 diabetes [16].

In the present day, the field of T1D research has witnessed a surge in the development of screening studies and preventive interventions. Researchers and healthcare organizations around the world are actively engaged in a multitude of approaches aimed at early T1D detection, the identification of high-risk individuals, and the implementation of preventive strategies designed to postpone or potentially arrest the disease's progression. Understanding and improving the utility of IAbs for T1D prediction is of paramount significance, yet it comes with 2 primary challenges and knowledge gaps. The first challenge lies in the substantial heterogeneity of T1D, accurately predicting disease progression from the appearances of IAbs to clinical T1D remains a complex task. The second challenge involves how to predict T1D at its earliest stage through IAbs. We will review the predictive features of IAbs, explore recent advances in identifying new IAbs and innovative methods for detecting IAbs to enhance the comprehension of IAbs as a biomarker for predicting T1D.

## Establishment of IAbs as the Standard Biomarkers for T1D Prediction

T1D is a polygenic disease, while genetic risk factors are necessary but not sufficient for the prediction. The concordance rate of T1D among monozygotic twins is reported to be lower than 30% [17, 18], although a recent study suggested that this percentage might be higher in long-term follow-up [19]. The major genetic risk factors are the HLA class II haplotypes, HLA-DR3-DQ2 and HLA-DR4-DQ8, which are found closely associated with the risk of development of IAbs [20, 21]. However, it has been observed in multiple studies that HLA plays quite limited roles for T1D progression among the subjects who have developed multiple IAbs [22, 23]. High-risk HLA haplotypes are found to be associated with the type and the age of first IAb seroconversion. Individuals with HLA-DR4-DQ8 are most likely to develop IAA first, with seroconversion peaking in the first years of life and declining over the following years [24, 25]. Whereas individuals with the HLA-DR3-DQ2 haplotype are more likely to develop GADA as a first IAb, with most frequent seroconversion occurring until the second year of life and remaining relatively constant [24, 26].

T lymphocytes are the central determinants for insulitis and β-cell destruction in T1D development. The role of T cells in T1D has motivated research consortium efforts to develop T-cell based biomarkers. Nevertheless, the challenge persists in T cell assays, characterized by significant variations in replicate samples measured across different laboratories [27, 28], resulting in a scarcity of robust and universally accepted disease biomarkers. Furthermore, evidence has recently been accumulating that the peripheral T cells are a different picture from the T cells within the inflammatory islets during insulitis [29], which complicates simply using the peripheral T cells as disease biomarkers reflecting the real T-cell repertoires of islet autoimmunity in the actual inflammatory islets.

Based on the discovery of IAbs, several birth cohort studies, including Diabetes Autoimmunity Study in the Young (DAISY), Type 1 Diabetes Prediction and Prevention (DIPP) study, BABYDIAB9, BABYDIET, and the Environmental Determinants of Diabetes in the Young (TEDDY), have been carried out over decades and have greatly enriched the understanding of natural history of T1D. These studies from different regions together documented the predictive value of IAb for T1D and the rate of progression from IAb seroconversion to clinical T1D [21]. Approximately 70% of individuals with ≥2 IAb progressed to clinical T1D within 10 years [21]. All these prospective birth cohort studies promoted the maturity of the theory of staging progression of T1D. In 2015, a scientific statement [16] was issued by the Juvenile Diabetes Research Foundation, the Endocrine Society, and the American Diabetes Association, defining 3 stages of T1D: stage 1 is characterized by the presence of 2 or more T1D associated IAbs; stage 2 includes individuals with 2 or more IAbs with progression to glucose intolerance or dysglycemia from loss of functional β-cell mass; and stage 3 is featured by the onset of typical clinical symptoms and signs of diabetes. The pathogenesis of T1D is a continuum but divided into stages starting with immunological abnormality marked by the appearance of IAbs, gradually progressing to metabolic dysfunction, and finally to symptomatic clinical diabetes with hyperglycemia. IAbs are well accepted to serve as the standard biomarkers for T1D progression.

### Stratifying T1D Progression Risk With Features of Islet Autoantibodies

While the number of IAbs is well established as being closely associated with the risk of T1D progression, in high-risk individuals with 2 or more IAbs, the progression of T1D can exhibit significant variability, ranging from a few days to more than 20 years. For individuals who have tested positive for IAbs through general population screening, what criteria should determine the need for close follow-up and initial intervention treatments like anti-CD3 therapy? Features of IAbs, such as IAb levels, age at first detection of IAbs, IAb types, number of IAbs, and IAb affinity, can provide valuable information about the likelihood and timeline of T1D development. Incorporating these features will lead to more accurate risk assessment and tailor preventive strategies accordingly.

#### Age and type of IAbs appearance

Evidence from birth cohort studies has shown that, in genetically at high-risk children, IAbs occur more frequently between the ages of 6 months and 3 years, with the peaking incidence at 9 months to 2 years of age [30]. Seroconversion at this early age was associated with a high rate of progression to diabetes and accounted for most children who developed clinical T1D by age 10 years [30]. The first appearance of IAb in children followed from birth is usually IAA or GADA, and subsequently IA-2A and/or ZnT8A, expanding from a single to multiple IAbs [20].

In the current general population T1D screening studies, the age and type of the first IAb appearance cannot be retrospectively determined. In TrialNet study participants with multiple IAbs (age range, 1-49 years), T1D risk decreased with the increase of ages at screening [31]. Among participants with 2 autoantibodies, those with GADA had less risk and those with IA-2A had higher risk of T1D. Participants with IAA and GADA had only a 17% 5-year risk of T1D, which was much lower than in genetically high-risk children [32]. In studies involving adult-onset T1D population, the IAb positivity decreased with increasing age [33]. GADA is the most frequent and dominant observed IAb in adults. Adults were less likely than children to be positive for one or more IAbs, with the majority having single GADA positivity (66%-78%) [33, 34]. However, the use of exogenous insulin among adult participants limited the detection of IAA, and there is currently a dearth of evidence regarding the presence of IAbs in adults at stage 1 or stage 2 of T1D. In adult patients following T1D onset, the levels of IAbs often decline, even turning negative [35, 36], which poses a challenge for the utility of IAbs in predicting or diagnosing T1D in adults.

#### Level of IAbs

Antibody levels may be informative and easy to stratify T1D risk. It has been noted in several studies that IAb levels are associated with T1D progression, but not for all the 4 IAbs, and the uncertain prediction hinders its direct clinical application. In studies of DAISY cohort, IAA level was one of the major determinants of the age of diabetes diagnosis [37]; lower initial IAA levels independently predicted slower progression to diabetes (>10 years) [38]. In the TEDDY study, higher IAA and IA-2A levels were associated with increased risk of diabetes in those children who were persistently autoantibody positive [32]. Recent studies of IAb levels take aim at characterizing IAb levels and improving its utility in T1D prediction. The Type 1 Diabetes Intelligence (T1DI) study combined data from 5 prospective cohort studies in Finland, Germany, Sweden, and the United States, with 1604 children with confirmed positivity, harmonized titers of IAA, GADA, and IA-2A. The study stratified the levels of IAb by quartiles and used the type-specific titer thresholds to identify children at-risk for T1D [39]. Further analysis showed that the T1D predictive power when using IAb levels alone was comparable with including IAb positivity and baseline covariates, and the prediction was better for shorter follow-up periods (≤2 years) and remained reasonable for longer follow-up periods (11 years) [40].

#### Number of IAbs

In long-term longitudinal birth cohorts, T1D risk by 15 years of age was 12.7%, 61.6%, and 79.1%, respectively, in genetically high-risk children with 1, 2, and 3 IAbs [21]. It is always in the common picture with a big jump of increasing risk from single IAb to multiple (2 or more) IAbs. Almost all the subjects with multiple IAbs will eventually develop clinical T1D with time, while the subjects with a single IAb only have a very low risk of progressing to T1D. While in the natural disease history, IAbs usually appear sequentially, beginning with a single IAb-positive, then to positive for 2 or more IAbs, before finally progressing to clinical T1D [41]; thus, the single IAb theoretically represents an earlier stage of higher risk with multiple IAbs. A study in T1D relatives (median age 16.2 years) demonstrated that relatives persistently positive for a single IAb had a 22% cumulative risk of progressing to multiple IAbs within 5 years; furthermore, the relatives who were converted to multiple IAbs from a single IAb during the follow-up had increased risk of developing clinical T1D compared with the relatives who tested positive with multiple IAbs at study entry [42]. In the Belgian Diabetes Registry study of 20 years follow-up of first-degree relatives of T1D, participants with a single IAb at baseline showed lower and slower progression to clinical onset compared with those with multiple IAbs (progression after 15 years: 30% vs 79%, progression after 20 years: 54% vs 88%) [22]. While a single IAb-positive result may not serve as a strong predictor, it is worth noting that some individuals with single IAb still progress to clinical T1D. The reduced predictive value of single IAb could potentially be attributed to low affinity or a lack of disease relevance.

#### Still IAbs

It has also been noticed that a small number of individuals with multiple autoantibodies showed slow or no progression to clinical T1D (more than 10 years after seroconversion), in which the autoimmunity seems “still.” In the DAISY study [38], older age at seroconversion and lower initial IAA levels independently predicted slower progression to diabetes in children with multiple IAbs. The SNAIL study [43] observed and reported these slow or nonprogressors from 5 longitudinal cohorts of individuals positive for multiple IAbs in the initial screening. The study found that the multiple IAbs in these nonprogressors could disappear during long-term follow-up; multiple IAbs converted to single IAb or complete negative. There were other reports of reversion to negativity from multiple IAbs, although they only happened in a small proportion [44, 45].

#### IAb affinity

The force of antibody-antigen binding is called *affinity* [46]. Antibody specificity for a given antigen is determined by its relative affinity. Previous studies have found that the IAbs with low affinity that are commonly seen in single IAb positivity are at low risk with less or non-disease relevance and this is consistent and well documented from multiple clinical studies [47-49]. Unlike the positivity of multiple IAbs that were usually persistent during disease progression until the time of overt clinical onset, the majority of single IAb disappeared during the follow-up with years, even months, behaving as “transient positivity,” and people with these single IAb were never progressed to the disease. High affinity IAA was associated with HLA DRB1*04, young age of IAA appearance, and subsequent progression to multiple IAbs or clinical T1D [47]. The affinity of GADA was heterogeneous, ranging from 10^7^ to 10^10^ L/mol [48]. GADA affinity was higher in multiple IAb-positive children and in HLA DR3-positive children, and lower in children with single GADA positive and/or GADA with specificities that were restricted to minor NH(2)-terminal GAD65 epitopes. Thus, in prospective studies, screening high affinity IAbs can increase the disease predictive value.

### Screening High Affinity IAbs With High Predictive Value

At initial screening either in first-degree relatives of patients with T1D or in the general population, up to 85% of instances of IAb positivity detected are single IAb [50], being single GADA or single IAA in most cases [32, 42]. However, most of single IAbs are low affinity, “transient” positivity with low disease risk [48, 51]. Continuing long-term follow-up for individuals with low affinity IAbs would consume significant manpower, material, and financial resources. Low affinity IAbs have the potential to result in the misdiagnosis of diabetes and may lead to misguided pre-T1D intervention efforts.

The IAb detection methodologies performed in these studies use the current gold standard radiobinding assay (RBA) technique that was reported to have high assay specificities, usually in the range of 98% to 99%. The current standard RBA, unfortunately, is not able to distinguish between high and low affinity autoantibodies unless absorption assay is performed, which is high-cost in terms of labor, time, and reagents. A recently developed nonradioactive IAb assay using an electrochemiluminescence (ECL) technology with its unique advantage of high affinity antibody detection can discriminate high affinity autoantibodies from low affinity autoantibodies and remove the positivity of low affinity signals generated by RBA. Prediction of disease risk for each IAb by ECL assay has been greatly improved without decreasing the sensitivity for detection of high-risk cases with multiple IAbs or truly pre-T1D who were followed to clinical disease. In DAISY, over 50% of the children with single IAb confirmed by ECL (n = 83) progressed to T1D in 10 years. In contrast, none of the 65 children who were single IAb-positive by RBA but negative by ECL progressed to diabetes [52]. In an ancillary study of TrialNet, the positive predictive values for clinical T1D of GADA and IAA by ECL assay were significantly increased over 50%, compared with RBA, from 15.7% to 23.8% (GADA, *P* < .0001) and 21.4% to 32.3% (IAA, *P* < .0001), respectively [53]. In this same cohort, the negative predictive values (reflex of the assay sensitivity) of ECL assay for both GADA and IAA were also shown to be significantly increased. In another ancillary study of TrialNet, subjects who were positive for a single IAb by RBA but negative by ECL showed no worsening of glycemia, similar to subjects negative for all IAbs, during a median follow-up period of 5.0 years [54]. In contrast, glycemia worsened significantly in the subjects with single IAb confirmed by ECL, comparably with the worsening in subjects with multiple IAbs; the latter group had a higher progression to T1D (30%). Both IA-2A and ZnT8A are usually thought to be not commonly seen as an isolated single IAb. However, in ongoing clinical screening in children of the general population, the Autoimmunity Screening for Kids (ASK) study, single ZnT8A was unexpectedly observed in nearly 50% among the children positive for ZnT8A by RBA, although overall ZnT8A positivity was much lower compared with GADA and IAA. Of these single ZnT8A detected by RBA, 86% were of low affinity, showing as negative by ECL assay, and had a low rate of progression to clinical T1D [49, 54]. High affinity IAbs at their very first initial positive visit will stay high affinity, consistent over time. Similarly, those low affinity at initial screening will stay low over time. No converting events from low to high or high to low affinity were seen over time [55, 56]. These results implied that high disease specific IAbs are capable of being pre-identified on the early stage of initial screening using a high affinity assay.

Unlike childhood T1D who were often seen with multiple IAbs at disease onset, a large proportion of adult-onset patients with T1D featured single positivity of GADA while the disease specificity and clinical value of GADA by current standard RBA remains questionable. In a study of 2 adult-onset T1D cohorts, the Action LADA and Diabetes in Young Adults (DiYA) studies, nearly 40% of single GADA positivity by RBA were shown negative by ECL assay, with low affinity, and their clinical phenotypes were more similar to type 2 diabetes [34]. High affinity ECL assay showed a greater clinical utility in screening adult-onset diabetes, by allowing for more accurate clinical diagnosis to the benefit of clinical care. In addition, GADA using N-terminus truncated GAD65 in RBA was also reported to improve the disease specificity by removing low affinity signals from antibodies binding to N-terminus [57].

## New Islet Autoantibody Assay Technologies for Current T1D Screening Studies

Although familial clustering is a common feature of T1D, with the risk of disease being up to 15-fold higher in families with T1D [58], the vast majority of children are diagnosed with sporadic cases of diabetes and the proportion of children with an affected first-degree relative at the time of diagnosis of T1D is only less than 10% [59, 60]. Therefore, the high-risk genotype cannot be a definitive predictor of the disease. General population screenings of IAbs for T1D prediction for public health have been started worldwide without consideration of genetic background. The Fr1da study in Germany [61], ASK in the United States [62], and several other studies [63] have been carried out for general population screening in children.

Mass screening of IAbs in the general population using the current standard RBA faces not only the problem of IAb affinity–associated disease specificity, but also the realistic challenge of cost-efficiency with the single assay platform, which is laborious, inefficient, and costly for measurement of 4 IAbs. There are currently 2 nonradioactive multiplexed assays that have been used for T1D screening in general populations, a combined enzyme-linked immunosorbent assay (ELISA) (3-Screen ICATM ELISA) in the German Fr1da study [61] and a multiplex ECL assay in the US ASK study (103). The 3-Screen ICATM ELISA combines 3 IAbs, GADA, IA-2A, and ZnT8A without IAA in one well [64] and the positive signals were then confirmed and identified by their corresponding single RBA. The multiplex ECL assay combines all 4 major IAbs and the autoantibodies to transglutaminase for celiac disease in one well to screen for the T1D and celiac disease simultaneously. The multiplex ECL assay also allows for the inclusion of more types of antibody assays, up to 10, to screen multiple autoimmune diseases simultaneously [65, 66]. The Fr1da study in Germany observed that the prevalence of high-risk with multiple IAbs in 90 632 children from 1.75 to 5.99 ages was 0.31% [46]. Of these high-risk children, the 3-year cumulative risk for stage 3 T1D was 24.9% (annualized rate, 9.0%). The prevalence of DKA in Fr1da study was 3.2% with milder onset symptoms, much lower than previously reported (20%∼40%) in unscreened children [67, 68], which demonstrates the prevention benefits of screening. The ASK study is an ongoing screening program. The study found IAb screening in the general population to be cost-effective [69] and well accepted by parents and providers [70]. From 32 366 children screened, the prevalence of high-risk with multiple IAbs was 0.53% and single high affinity IAb (positive by both RBA and ECL) added another 0.56% (unpublished data). The prevalence of DKA was also found to be very low in the ASK study, only 5% so far among small number of children who progressed to clinical T1D (unpublished data). Another multiplex IAb assay, antibody detection by agglutination-PCR (ADAP), has been recently reported which combines all 4 IAbs and transglutaminase and this multiplex assay was tested in new-onset T1D children with comparable sensitivity [71]. In addition, luciferase immunoprecipitation system (LIPS) has been reported to have comparable performance with RBA in GADA and IA-2A [72], high-performance on IAA and improved discrimination of progressors to T1D in false discovery rate compared with RBA [73]. The advantages and the limitations of current available assay platforms are summarized in Table 1.

**Table 1.** Comparison of IAb assays

Assay name	Type of IAbs detected	Advantages	Limitations
RBA	GADA, IAA, IA-2A, ZnT8A	Stable, widely used in research studies and clinics	Radioactive, high labor-cost, single antibody assay
ECL	GADA, IAA, IA-2A, ZnT8A	High efficiency, high affinity, nonradioactive, low cost, low serum consumption	Requirement for special equipment
Bridge-ELISA	GADA, IA-2A, ZnT8A	High efficiency, commercialized, nonradioactive	Lack of IAA assay, need RBA to identify positive IAb types
ADAP	GADA, IAA, IA-2A, ZnT8A	High efficiency, nonradioactive, low serum consumption	Lack of evidence in pre-T1D studies
LIPS	GADA, IAA, IA-2A, ZnT8A	High sensitivity, nonradioactive, low serum consumption	Single antibody assay, lack of evidence in pre-T1D studies

Abbreviations: ADAP, antibody detection by agglutination-PCR; ECL, electrochemiluminescence; ELISA, enzyme-linked immunosorbent assay; GADA, autoantibody to glutamic acid decarboxylase 65; IA-2A, autoantibody to insulinoma-associated antigen-2; IAA, autoantibody to insulin; IAb, islet autoantibody; LIPS, luciferase immunoprecipitation system; RBA, radiobinding assay; T1D, type 1 diabetes; ZnT8A, autoantibody to zinc transporter 8.

## Explore New Islet Autoantibodies to Improve Prediction

While GADA, IAA, IA-2A, and ZnT8A are the most well-known IAbs associated with T1D, researchers are investigating other potential autoantibodies to improve early prediction and prediction accuracy. By expanding the panel of autoantibodies, it may be possible to detect T1D even in cases where the traditional markers are not present.

### Autoantibodies to the Extracellular Domains of IA-2 and ZnT8

**Figure 1.**
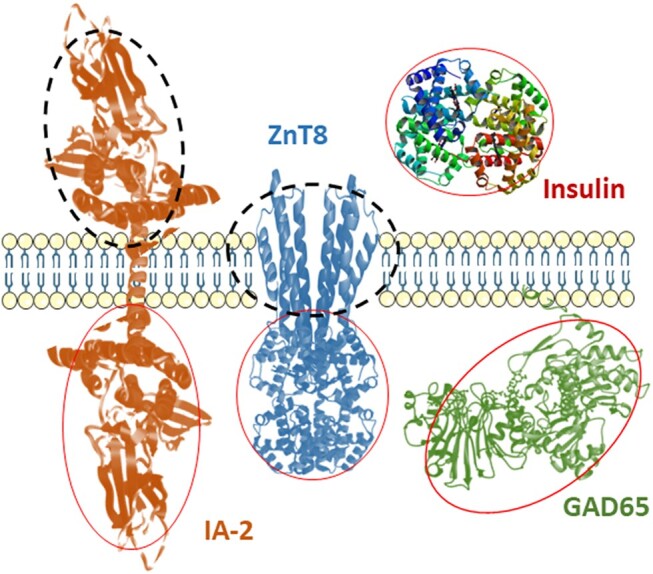
Four major biochemical islet autoantigens, insulin (IAA), glutamic acid decarboxylase 65 (GAD65), insulinoma-associated antigen-2 (IA-2), and zinc transporter 8 (ZnT8), in relation to the cell membrane. Red and black circles mark the domains that are used or not used, respectively, in standard islet autoantibody assays. Currently most laboratories are using full-length GAD65 for GADA detection, insulin or proinsulin for IAA, IA-2 intracellular domain for IA-2A, and ZnT8 intracellular domain for ZnT8A detection (in red circles). Autoantibodies against the extracellular domain of IA-2 or transmembrane domain of ZnT8 (in black circles) have been reported yet need more data to verify their roles in T1D diagnosis and prediction.

Both IA-2 and ZnT8 are the membrane proteins spanning the insulin secretory granules of the β-cells. The current IA-2A and ZnT8A widely applied in all laboratories are the autoantibodies to intracellular domains of IA-2 (IA2ic) and ZnT8 (ZnT8ic) (Fig. 1). Since the major epitopes of autoantibodies to IA-2 were found in the protein tyrosine phosphatase region of the IA-2 molecule within the intracellular domain and the assay of autoantibodies to full-length IA-2 was resulted in a high background, the intracellular domain of IA-2 (IA2ic) has been widely accepted to be used in the IA-2A test. ZnT8 is a 2-modular membrane protein consisting of a 6-spanning membrane domain and a C-terminal intracellular domain. Detection of autoantibodies to extracellular epitopes of ZnT8 (ZnT8ec) is quite challenging because of technical difficulties in presentation of a natively folded full-length ZnT8 antigen and adaption of the insoluble 6-spanning membrane regions to a solution-based assay platform [74].

Studies of autoantibodies targeting the IA-2ec domain have been addressed again in recent years [75]. Autoantibodies targeting the IA-2ec domain (IA2ecA) can be detected in patients with T1D and in a subgroup of adult-onset diabetes negative for conventional IAbs and clinically diagnosed as type 2 diabetes. More recent studies have shown that IA2ecA can be detected in overall 17% (185/1072) of patients with newly diagnosed T1D, including children and adults [76] (including unpublished data). The IA2ecA was detected in patients either positive or negative for IA2icA, suggesting that IA2ecA is independent of IA2icA positivity. Further study in the pre-T1D subjects showed that only 2.6% were positive (unpublished data). In another study, antibodies reacting with a variant full-length IA-2 molecule (with 4 single nucleotide polymorphisms) was found to be associated with accelerated progression to T1D in those positive for GADA and/or IAA but negative for IA-2A [77]. The study claims that molecular modeling of IA-2 that alters 3 amino acids induces changes in the 3-dimensional structure of the molecule, which may lead to epitope unmasking in the IA-2ec domain.

Very recently, the autoantibodies to ZnT8ec (ZnT8ecA) have been identified in patients with T1D and in pre-T1D subjects [78, 79]. Remarkably, ZnT8ecA from T1D patients was demonstrated to bind on the surface of live β-cells and this is the first biochemically identified β-cell surface IAb present in patients with T1D. Islet cell-surface autoantibodies (ICSAs) in T1D were first described in the 1970s [80] and were examined for lytic effects on pancreatic β-cells [81], but ICSAs have never been defined biochemically, and the molecular target of putative ICSAs remained unknown until this recent study. The study further investigated the timing of seroconversions of ZnT8ecA and its relationship with 4 established IAbs [79]. From 30 selected children who were followed from birth and sequentially developed 4 major IAbs before clinical T1D onset, 10 tested positive for ZnT8ecA. Of these 10 children positive for ZnT8ecA, all had detected ZnT8ecA as the first seroconverted IAb, including 7 before IAA and/or GADA and 3 who had detected ZnT8ecA, IAA, and/or GADA at the same time. The onsets of ZnT8icA exhibited a median delay of 4.3 years than ZnT8ecA, reflecting an intramolecular epitope spreading event from extra- to intracellular sites.

### Antibodies to Posttranslationally Modified Islet Antigens

Autoimmune reactivity to posttranslational modification (PTM) of antigen molecules has attracted much attention in T1D studies recently. PTM can contribute to autoimmune etiology at many levels [82]. At the level of peptides, PTM leads to altered sequences that are recognized with increased affinity. At the level of proteins, PTM can alter structure and subsequent processing by antigen-presenting cells. At the level of cellular processes, PTM can alter signaling pathways, leading to modified biological function. A bunch of studies have showed significant increased T-cell recognition for PTM neoantigens or neoepitopes in T1D, such as oxidized proinsulin [83], deamidated preproinsulin, ZnT8, IA-2, GAD65 [84], crosslinked chromogranin A [85], and so forth. The studies of autoantibodies on these PTM antigen molecules are still very preliminary in T1D. Hybrid insulin peptides (HIPs) have been found to exist in both mice and human β-cells [86] and stimulate CD4 T cells from NOD mice and T1D patients [87]. The antibodies to HIPs were recently identified in patients with T1D [88] and these HIP antibodies could appear very early in T1D development, even preceding all known IAbs, including IAA. Unfortunately, HIP antibodies were also found in healthy control subjects with similar levels and frequencies and so they are not able to play a role in differentiating T1D patients from healthy subjects. Adapted from a human T-cell study on a PTM-IA2ec molecule with point mutations by deamidation (Q > E) in 4 locations [75], novel autoantibodies to this PTM-IA2ec were identified in a large proportion of new-onset T1D children [76]. These autoantibodies to PTM-IA2ec are independent of the prototypical IA-2A and superior to the detection of wide-type IA2ec autoantibodies in frequency [76]. However, similar to wide-type IA2ecA, PTM-IA2ecA is not commonly seen in the pre-T1D stage (unpublished data). In recent years, a study group reported the autoantibodies to oxidized insulin in patients with T1D [89]. Further studies, especially in subjects at pre-T1D stages, will be expected to verify the predictive values for these oxidized insulin autoantibodies.

## Future Perspectives

Although substantial progress has been achieved, the early prediction of T1D continues to evolve. High affinity single IAb would probably be able to predict T1D at an earlier stage. The presymptomatic early recognition of T1D would benefit the patients with lower DKA incidence, milder onset symptoms, and lower medical cost. More promisingly, once screened with stage 1 of T1D, symptomatic T1D can be delayed by immunotherapy [90]. Multiplex IAb assay platforms should have the potential to incorporate high affinity, high efficiency, and low cost, which is necessary for large-scale general population screening of T1D. PTM islet neoepitopes/neoantigens may generate new autoantibodies or, alternatively, increase affinity of autoantibody binding that may help the T1D prediction. These novel candidate autoantibodies need more evidence in studies of pre-T1D population to verify their roles in T1D prediction. With the combination of genomic, transcriptomic, and proteomic technologies, integrating multiple biomarkers with islet autoantibodies, such as genetic markers, metabolic indicators, may provide more comprehensive and refined prediction models serving for early prediction and intervention of T1D.

## Data Availability

Original data generated and analyzed during this study are included in this published article.

## Abbreviations

ASK: Autoimmunity Screening for Kids
DAISY: Diabetes Autoimmunity Study in the Young
DKA: diabetic ketoacidosis
ECL: electrochemiluminescence
ELISA: enzyme-linked immunosorbent assay
GADA: glutamate decarboxylase autoantibodies
HIP: hybrid insulin peptide
IA-2A: insulinoma-associated antigen-2 autoantibodies
IAA: anti-insulin autoantibodies
IAb: islet autoantibody
ICSA: islet cell-surface autoantibodies
PTM: posttranslational modification
RBA: radiobinding assay
T1D: type 1 diabetes
TEDDY: The Environmental Determinants of Diabetes in the Young study
ZnT8A: zinc transporter 8 autoantibodies
